# U.S. policy research funded by the National Institute of Mental Health, 1993-2024

**DOI:** 10.1093/haschl/qxag099

**Published:** 2026-04-29

**Authors:** Briana S Last, Nicole Teow, Madeline Poupard

**Affiliations:** Department of Psychology, Stony Brook University, Stony Brook, NY 11794, United States; Department of Psychology, Stony Brook University, Stony Brook, NY 11794, United States; Department of Psychology, Stony Brook University, Stony Brook, NY 11794, United States

**Keywords:** federal funding, policy research, mental health

## Abstract

**Introduction:**

We sought to identify the share of National Institute of Mental Health (NIMH) funding allocated to U.S. policy research over the past 3 decades (1993-2024).

**Methods:**

We systematically searched the National Institutes of Health Research Portfolio Online Reporting Tools Expenditures and Results database using keywords. Manually reviewing the titles, abstracts, and publications of all projects identified through this search, we categorized a project as U.S. policy research if it studied the outcome(s) and/or implementation of a policy and if it had a mental health focus. We measured the share of NIMH projects and funding allocated to policy research as a percentage of all NIMH projects and funding between 1993 and 2024. We also examined whether NIMH projects and funding for policy research changed between 1993 and 2024.

**Results:**

Aggregating all NIMH funding between 1993 and 2024, NIMH funded 110 969 projects and allocated $39.34 billion to research, of which 1510 projects (1.36%) and $800.59 million (2.04%) were policy research. NIMH funded 29 policy projects ($16.27 million) in 1993 and 62 policy projects ($33.69 million) in 2024, a 113.79% increase in policy projects.

**Conclusion:**

NIMH allocates a small share of research dollars and projects to U.S. policy research despite policies' importance to population mental health.

Key points

- Growing evidence suggests that U.S. policies impact population mental health, yet the extent to which the National Institute of Mental Health (NIMH) funds policy research is unclear.
- By examining the last 3 decades of NIMH funding, the authors found that NIMH allocates a small share of research funding to U.S. policy projects.

U.S. federal, state, and local governmental policies shape the mental health and well-being of Americans.^1-3^ Policies can impact mental health directly by targeting people's access to mental health prevention and intervention services and indirectly by targeting people's access to material and social resources.^4-6^

Robust evidence suggests that policies that directly increase mental health treatment access not only increase treatment availability and utilization, but also improve population mental health outcomes.^7^ For example, between 2014 and 2017, states that had expanded Medicaid had about 293 fewer suicide deaths per year than non-expansion states.^8^ Policies that do not directly target mental health but reduce economic inequality and poverty also have population mental health effects. For example, the Earned Income Tax Credit (a tax credit for working parents) is associated with decreasing married mothers' depression symptoms by 15.7% and increasing their self-esteem by 10.1%.^9^ Conversely, growing evidence suggests that policies restricting the rights of immigrants, LGBTQIA+ people, and the economically vulnerable are having negative population mental health ramifications.^2,10,11^ A recent study found that the enactment of state anti-transgender laws between 2018 and 2022 was associated with a 7%-72% increase in past-year suicide attempts among transgender and nonbinary young people.^2^

Despite growing recognition and evidence that policies have robust impacts on population mental health, the mental health field remains largely focused on researching individual factors that shape and improve mental health.^1,7,12^ The National Institute of Mental Health (NIMH), the largest funder of mental health research in the United States (U.S.)^13^ has, for the past several decades, made significant investments in understanding the neurobiological and genetic correlates of psychiatric disorders.^14-16^ This focus on basic biological science relative to applied intervention research led one former NIMH director to reevaluate his role in shaping the agency's investments in biological psychiatry.^17^ Researchers, professional organizations, and policymakers have critiqued the institute for the decades-long decline in investments in clinical and services research.^18-20^ Yet, less attention has been paid to NIMH's investments in policy research, despite growing calls from within the mental health field for research spending to reflect the role of policies in shaping psychological health.^12,21^

Determining the optimal amount of NIMH funding dedicated to policy research first requires understanding how much the institute currently spends on policy research. To our knowledge, there has not yet been an analysis of how much of NIMH's research spending is allocated to policy research or whether NIMH's investments in policy research have changed over time. The objective of the present study was to investigate this question by: (1) examining the share of NIMH projects and dollars dedicated to policy research; and (2) tracking whether the amount of NIMH-funded policy research has changed over the last 3 decades.

## Data and methods

### Data extraction

Using similar methods described elsewhere,^18,22,23^ we systematically searched the National Institutes of Health (NIH) Research Portfolio Online Reporting Tool (RePORTER)—a publicly available database of all NIH funding—to identify all projects that received NIMH funding between 1993 and 2024. We selected 1993 as the starting point because it was the first full fiscal year that NIMH rejoined the NIH through the Alcohol, Drug Abuse, and Mental Health Administration (ADAMHA) Reorganization Act.^24^ This Act reorganized the federal government's agencies such that the ADMHA's dual functions of services and research were split, with NIMH dedicated to research, and the newly formed Substance Abuse and Mental Health Services Administration dedicated to services.^16^ We ended our search in 2024 given NIH's uncharacteristic year in 2025.^25^

Informed by a similar search strategy employed elsewhere,^22^ we conducted an advanced text search in RePORTER, assessing whether any of the following keywords appeared in an NIMH project title, terms, or abstract: policy, policies, law, legal, waiver, payment, pay, reimbursement, Medicare, Medicaid, statute, “executive order,” or bill. All NIMH RePORTER projects funded between the fiscal years of 1993 and 2024 identified through this search strategy were exported to a spreadsheet. In some cases, the exported RePORTER spreadsheet was missing project and funding information available on the website; these data were manually added to the spreadsheet to ensure accuracy. This spreadsheet constituted the study's final dataset, which our team manually coded to identify U.S. policy projects (Figure S1).

### Coding

Using the final dataset, our team of 3 coders reviewed each project title, abstract, and the project's associated publications (primarily identified through RePORTER) to determine if the project was U.S. policy related. We followed NIH's grant number system to categorize projects.^26^ Each project is designated by a project number, corresponding to one fiscal year and/or one aspect of a grant.^26^ NIH defines a “new” project as a “Type 1” application (ie, a project receiving funding for the first time).^27^ It is a proxy for unique NIH grants because multi-year grants are only counted once—in the initial year of funding.^26^ For example, our keyword search identified the following 3 projects: 5R01MH046933-02, 3R01MH046933-02S1, and 3R01MH046933-02S2. Each project is part of the grant, “Mental health care payment options under Medicare Part B,” which studied the effects of changes to Medicare's coverage of mental health services, including changes enacted by the 1989 Omnibus Budget Reconciliation Act. Notably, the first year (“Type 1”) of the grant (1R01MH046933-01A1) was not included in the dataset because that project was awarded in 1992, the year before our search inclusion criteria. As such, this grant was associated with three projects and no “new” projects in our study. Notably, this grant included 2 years of administrative supplement projects, which shared the abstract of the parent grant in RePORTER (a common RePORTER feature) and therefore received the same code as the parent grant in our coding scheme (ie, all projects associated with this grant were coded as U.S. policy projects).

We coded a project as “U.S. policy research” if the project studied the impact and/or implementation of local, city, state, tribal, and/or federal policy. After consulting a political scientist and the policy literature, we decided to use American political scientists Karen Orren's and Stephen Skowronek's conceptualization of what a policy is, as elaborated in their book *The Policy State*.^28^ They define a policy as a “commitment to a designated goal or course of action, made authoritatively on behalf of a given entity or collectivity, and accompanied by guidelines for its accomplishment.”^28^ Per this definition, we coded the following project as policy research (1R01MH097298-01A1, “The Effects of State and Federal Insurance Policies on Quality of Care for Autism”) because the project examined how state legislation and Medicaid waivers were associated with access to care for autistic children and their families. Several mental health services projects, while very policy-relevant, ultimately were not coded as U.S. policy research. For example, we coded the following project (5R01MH047345-02, “Mental health service utilization by foster children”) as non-policy research. Although identifying foster children's mental health utilization patterns may be distally attributable to some federal, state, and local policies, and even though the researchers planned to synthesize their findings for policymakers, the study did not directly examine the effects and/or implementation of a specific policy or set of policies.

We excluded projects that did not have a mental health focus and that were conducted outside of the United States and/or studied non-U.S. policies given our interest in identifying research that has the potential to influence U.S. policymaking. We considered a project as focusing on mental health if it studied a mental health policy (eg, 5R01MH046933-02 described above) and/or if it examined the mental health effects of a policy regardless of whether the policy specifically targeted mental health. For example, we considered the following project U.S. policy research with a mental health focus: 1R03MH128649-01 (“Inequalities in Mental Health During the COVID-19 Pandemic: The Interplay of Individual Pandemic Stressors and State Sociopolitical Contexts”), a project that studied how state-level public health emergency policies enacted during the coronavirus pandemic affected constituents' mental health. Notably, for historical, institutional, and disease-specific reasons, the NIMH funds a substantial amount of HIV research;^29^ this research does not always have an explicit mental health focus. For instance, the following HIV project (1K05MH001376-01, “Ethical and policy issues in the AIDS epidemic”), while policy-relevant, did not appear to have a mental health focus.

To ensure consistent application of the policy definition when coding, we engaged in a multi-step, iterative team coding process. First, all 3 coders met as a team to discuss the operationalization of the “policy research” definition and to review how the team would code projects in the final data set. All 3 team members independently double-coded 106 projects, met to discuss our codes, and further refined our operationalization. One coder created a codebook with this operationalization of policy research, which the other coders reviewed and refined. Then, 2 coders independently double-coded 303 projects and achieved 96.4% agreement (Cohen's kappa = 0.82). Our team met to discuss discrepancies and reached consensus on all disagreements for this initial set. Given the high level of coder agreement and reliability, the same 2 coders independently single-coded the remaining projects. During this independent coding process, coders were encouraged to err on the side of caution and request a second opinion from the other coder regarding any uncertainties. In the event where the outcome remained unclear, the 2 coders consulted the third member of our coding team. Our team of 3 coders met regularly to resolve discrepancies via consensus discussion and to review coding questions and uncertainties. Once we coded all projects in the final dataset, we spot-checked projects that were coded early in the coding process to ensure consistent code application.

### Analysis plan

We characterized NIMH-funded U.S. policy research in both absolute and relative terms. In absolute terms, we calculated the total and new (ie, “Type 1”) U.S. policy projects and dollars allocated to these projects by NIMH. We used the “Total Costs by IC [Institute/Center]” data from RePORTER to identify NIMH's spending on each policy project, not the total costs of the grant (which may include contributions from other institutes). In relative terms, we calculated the share of policy research projects and funding as a percentage of total NIMH projects and funding (“Total Costs by IC”). Finally, we tabulated these absolute and relative statistics in aggregate (ie, over the 1993-2024 study period), by fiscal year, and by activity code.

To demonstrate whether NIMH-funded policy research funding changed over the 3-decade period under study, we calculated the percentage difference in policy funding from 1993 to 2024. We calculated this percentage difference in terms of project count and dollar amount. In addition, we also adjusted all funding amounts to constant “2024 dollars” using the NIH Biomedical Research and Development Price Index (BRDPI)^30^ as has been done in prior research examining NIH funding.^23^ These BRDPI-adjusted dollars enable researchers to analyze “real” funding investments over time, accounting for inflation in biomedical research costs.

We also analyzed policy research funding for Cooperative Agreements (U), Program Project/Center Grants (P), and Training Grants (T) separately. These grant mechanisms tend to have a broad scope and involve multiple subprojects (which, in our study, were all coded under a single parent project umbrella). If one aspect (including a publication) or subproject of a U, P, or T grant was considered U.S. policy research, the entire parent project was coded as a policy research project, which risks overestimating the amount of NIMH dollars dedicated to policy funding. To avoid overestimating policy research funding, we present funding analyses both with and without the inclusion of U, P, and T grants.

## Study results

### Total and new policy research funding

Aggregating all NIMH funding between the fiscal years 1993 and 2024, NIMH funded 110 969 total projects or 28 604 new projects, of which 1510 total projects (1.36%) or 275 new projects (0.96%) were U.S. policy research projects. Excluding cooperative agreements, center, and training grants (U, P, and T grants) from the policy counts, NIMH funded 784 policy projects (0.71% of total NIMH projects) or 234 new policy projects (0.82% of total new NIMH projects). Between the years 1993 and 2024, NIMH funded $39 335 441 793 in research and $800 592 650 in policy research (2.04% of total NIMH funding). Excluding U, P, and T grants from policy research funding, NIMH funded $270 649 487 (0.69% of total NIMH funding) in policy projects. The institute funded $10 875 665 607 in new projects and $123 853 151 in new policy research (1.14% of new NIMH funding) and $88 153 337 (0.81%) in new policy research when excluding U, P, and T grants. Adjusting dollar amounts to 2024 dollars per the BRDPI, total NIMH funding amounted to $57 290 801 695 and NIMH funding for policy research amounted to $1 266 274 379 (2.21%) of funding including U, P, and T grants and $394 731 302 (0.69%) excluding them. Real (ie, BRDPI-adjusted) new NIMH funding amounted to $14 811 196 716 and new NIMH funding for policy research amounted to $183 935 967 (1.24%) of funding including U, P, and T grants and $122 087 815 (0.82%) excluding them (Table 1 and Table S1).

**Table 1:** National Institute of Mental Health (NIMH) funding for U.S. policy research in projects and dollars, 1993-2024.

	National Institute of Mental Health (NIMH)	Policy research	Policy Research excluding UPT projects
**Total Projects (*n*)**	110 969	1510	784
Share of NIMH Projects (%)	100.00%	1.36%	0.71%
Annual Average	3468	47	25
Project Growth, 1993-2024 (%)^a^	43.82%	113.79%	238.46%
**New Projects (*n*)**	28 604	275	234
Share of New NIMH Projects (%)	100.00%	0.96%	0.82%
Annual Average	894	9	7
Project Growth, 1993-2024 (%)^a^	40.21%	200.00%	350.00%
**Total Projects Funding ($, unadjusted)**	$39 335 441 793	$800 592 650	$270 649 487
Share of NIMH Projects (%)	100.00%	2.04%	0.69%
Annual Average	$1 229 232 556	$25 018 520	$8 457 796
Funding Growth, 1993-2024 (%)^a^	357.48%	107.06%	453.33%
**New Projects Funding ($, unadjusted)**	$10 875 665 607	$123 853 151	$88 153 337
Share of New NIMH Projects (%)	100.00%	1.14%	0.81%
Annual Average	$339 864 550	$3 870 411	$2 754 792
Funding Growth, 1993-2024 (%)^a^	1238.01%	368.59%	637.37%
**Total Projects Funding ($2024, Adjusted)^b^**	$57 290 801 695	$1 266 274 379	$394 731 302
Share of NIMH Projects (%)	100.00%	2.21%	0.69%
Annual Average	$1 790 337 553	$39 571 074	$12 335 353
Funding Growth, 1993-2024 (%)^a^	78.62%	−19.16%	116.04%
**New Projects Funding ($2024, Adjusted)^b^**	$14 811 196 716	$183 935 967	$122 087 815
Share of New NIMH Projects (%)	100.00%	1.24%	0.82%
Annual Average	$462 849 897	$5 747 999	$3 815 244
Funding Growth, 1993-2024 (%)^a^	422.40%	82.95%	187.89%

Source: Authors' analysis of publicly available data from National Institutes of Health (NIH) Research Portfolio Online Reporting Tools Expenditures and Results (RePORTER).

^a^Growth is defined as the percentage difference in the number of projects (*n*) or funding ($) between fiscal years 1993 and 2024.

^b^Dollar amounts are adjusted to 2024 dollars according to the National Institutes of Health Biomedical Research and Development Price Index. Source: https://officeofbudget.od.nih.gov/gbipriceindexes.html.

### Changes in U.S. policy research funding, 1993-2024

**Figure 1:**
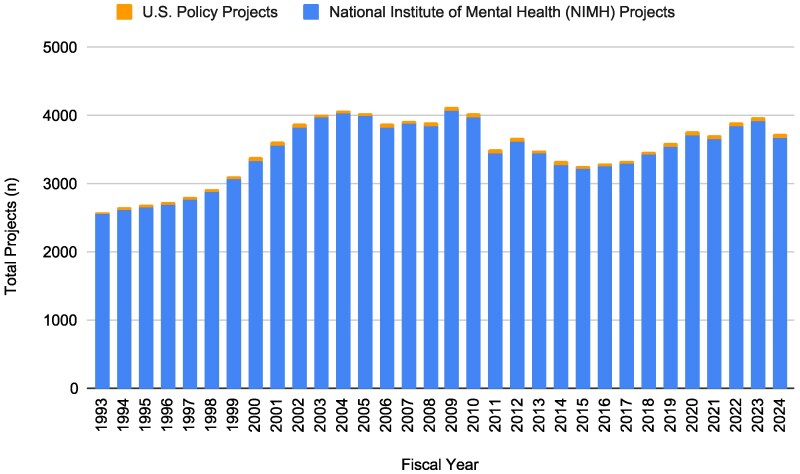
National Institute of Mental Health (NIMH) funding for policy research over time by project count, 1993-2024. Source: Authors' analysis of publicly available data from NIH RePORTER.

The number of NIMH policy research projects increased between 1993 and 2024. In 1993, NIMH funded 29 policy research projects (1.13% of the 2558 total NIMH-funded research projects that year) and in 2024, NIMH funded 62 policy projects (1.69% of the 3679 total NIMH-funded research projects that year). The absolute difference between the number of policy projects funded in 1993 and in 2024 represents a 113.79% increase in NIMH-funded policy projects. Calculating the change in NIMH-funded policy projects as a share of total NIMH projects, the change from 1.13% to 1.69% of NIMH projects between 1993 and 2024 represents a 48.65% increase in the policy-to-total NIMH project ratio. Notably, the growth in the number of policy projects between 1993 and 2024 exceeded the growth in all NIMH projects during this period. NIMH funded 2558 total projects in 1993 and 3679 total projects in 2024, which represents a 43.82% increase in the number of NIMH projects funded in the first and last fiscal year of the study period (Table 1 and Figure 1).

NIMH funding for policy research increased between 1993 and 2024 in unadjusted dollars. In 1993, NIMH funded $16 270 353 in policy research and in 2024, the institute funded $33 689 276 in policy research, representing a 107.06% increase in policy research dollars between 1993 and 2024. In 2024 dollars, NIMH funded $41 672 659 in policy research in 1993, and in 2024, it funded $33 689 276 in policy research. This decrease in real policy research funding represents a 19.16% contraction in constant 2024 dollars while real total NIMH spending grew during this time period. In 2024 dollars, total NIMH funding went from $1 152 579 490 in 1993 to $2 058 699 815 in 2024, which represents an 78.62% growth in funding. Thus, between 1993 and 2024, real spending (adjusted for 2024 dollars) on policy projects was outpaced by growth in total NIMH spending.

**Figure 2:**
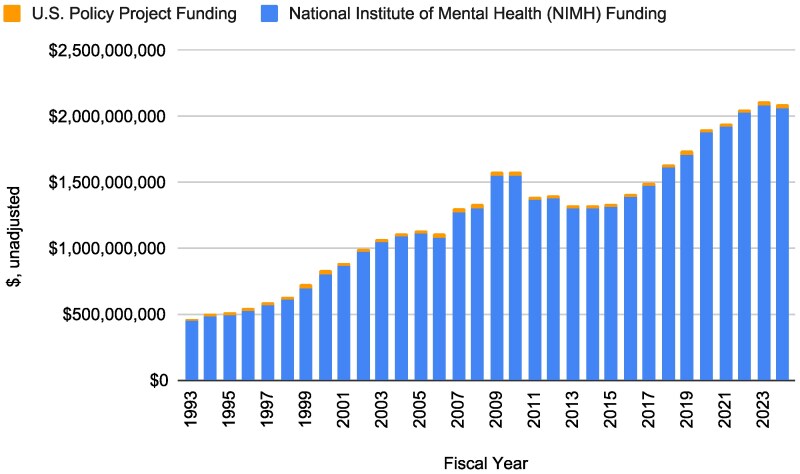
National Institute of Mental Health (NIMH) funding for policy research over time in unadjusted dollars, 1993-2024. Source: Authors' analysis of publicly available data from NIH RePORTER.

Finally, calculating the change in NIMH policy funding as a share of total NIMH funding (ie, the policy-to-total NIMH funding ratio), the change from 3.62% of NIMH dollars in 1993 to 1.64% of NIMH dollars in 2024 represents a 54.74% decrease in NIMH dollars spent on policy research as a share of total NIMH funding. Notably, for both the unadjusted and adjusted funding analyses, new policy and non-U, P, T policy research funding grew, with funding for these policy projects increasing in recent years. That is, in 1993, new policy project funding (in 2024-adjusted dollars) was $5 068 802 ($3 221 137 without U, P, T funding) and $9 273 429 in 2024 (entirely comprised of non-U, P, or T funding), which represents a 82.95% increase in new policy research spending (and a 187.89% in new non-U,P,T funding) during this time period. Thus, any observed relative declines in policy funding were largely observed for U, P, and T policy grants (Table 1 and Figure 2; Figures S2-S4).

## Discussion

Between 1993 and 2024, U.S. policy research has remained a small share of total NIMH funding. Approximately 1% of NIMH projects and 2% of NIMH dollars have been dedicated to policy research. Despite the small share of NIMH funding allocated to policy research, between 1993 and 2024, NIMH funded more policy projects, with the number of policy projects increasing by 113.79%, outpacing the growth in total NIMH projects during the same period, which increased by 43.82%. However, analyzing funding in dollars for NIMH policy projects over this period paints a more complex picture. Total spending (unadjusted and adjusted for inflation) in policy projects lagged behind growth in NIMH spending overall. Altogether, although the number of NIMH-funded policy projects grew in the past 3 decades, spending on policy projects did not keep pace with NIMH's spending growth and remains a small portion of NIMH research portfolio.

These findings reveal a potential disjuncture between growing evidence from scientific research on the importance of policies shaping health outcomes and public funding for scientific research. Although there is increasing recognition and evidence that policies, and social determinants more broadly, shape mental health and well-being,^1,7^ NIMH appears to invest only a small share of its dollars in policy research with a mental health focus. Given that NIH institute leaders have historically been drawn from the scientific community, it is possible that these funding allocations reflect the interests and priorities of mental health researchers. Indeed, as many scholars have documented, the U.S. mental health field has tended to focus its efforts on examining individual-level causes and interventions for psychological distress at the expense of studying structural determinants and solutions.^12,31,32^ The RePORTER database only contains funded projects, thus we do not have information on submitted but unfunded NIMH policy grant proposals. Analyzing these proposals might help identify whether NIMH spending reflects scientists' “bottom-up” priorities or NIMH's “top-down” agenda setting—with the caveat that investigators are unlikely to submit grants with no chance of funding.

Existing survey data suggest that both researchers and the public would welcome more federal investments in the social determinants of mental health as well as policy research that investigates mental health service quality, prevention, early detection, and health equity.^33-35^ Although more evidence directly examining the funding priorities of the U.S. public and scientists is needed to determine the appropriate level of NIMH funding for policy research, based on existing evidence, it is likely that NIMH's current policy research investments lag behind scientific and public consensus regarding the importance of studying the policy determinants of mental health. As the country's largest mental health funder, NIMH plays a critical role in what research is conducted in the U.S. and the institute's allocation of its research dollars also invariably shapes how the causes of and solutions to mental health challenges are conceptualized. NIMH's low investment in policy research may not only lead to a lack of understanding of how policies can shape the distribution and alleviation of mental illness, but it may also perpetuate current government disinvestment in policies and programs that can support population well-being.^7,12,35,36^

It is possible that other NIH institutes (eg, the National Institute on Drug Abuse, the Agency for Healthcare Research and Quality) may invest more in policy research, which could mitigate some of these concerns. Future research should examine the extent to which all NIH institutes fund mental health policy research. Regardless, given the unique mandate of NIMH, it is likely that other institutes cannot comprehensively and sufficiently fund all mental health policy research, suggesting that our findings still raise questions about the level of NIMH investment in policy research. The NIMH and other NIH institutes may be hesitant to fund policy research because these projects may be perceived to jeopardize NIH's bipartisan character. An in-depth characterization of NIH-funded policy research projects, including an analysis of the content of the NIMH-funded policy research identified in the current study, may shed light on this possible explanation for the low levels of policy research funding. This research could examine the kind of U.S. policy research NIMH funds (eg, which branches of government, governmental jurisdictions, policy levers, diseases, populations, etc.) to understand the scope and content of the institute's research portfolio.

### Limitations

There are several limitations to this study. First, our study's scope was intentionally focused on projects that examined policies' effects or implementation. Thus, training grants, fellowships, and early career awards that did not research policies and only offered researchers policy training (eg, didactics, coursework) did not count as policy research projects in our study. That is, we characterized NIMH's investments in policy research projects, not the institute's general policy focus. Second, despite our efforts to comprehensively identify all NIMH-funded U.S. policy research (eg, keyword search, project publication review), project abstracts (only 30 lines) provide limited information. Further, recently funded projects may lack corresponding publications, thus we may have underrepresented more recently funded policy projects. Third, although RePORTER is the best available tool to track NIH funding, it also comes with several methodological limitations. As we described previously, supplemental project abstracts did not consistently have unique abstracts from their parent projects in RePORTER, and thus we could not assess these projects separately. Additionally, RePORTER-exported datasets occasionally had missing information that required manual data entry, which is prone to human error.

Fourth, we investigated funding by one institute and country to limit the scope of our search to the largest funder of mental health research in the U.S. NIMH's U.S. policy funding allocations may not generalize to other institutes, funders, or countries. Finally, we investigated NIMH funding between 1993 and 2024, and ended our search just before large cuts and grant terminations were instituted across NIH.^25^ The future budget, organization, and research priorities of NIMH, and NIH more broadly, are uncertain. It is therefore unclear whether our findings will generalize as transformations to NIH unfold.

## Conclusion

Over the past 3 decades, NIMH—the largest funder of mental health research in the country—has dedicated a small share of its funding to policy research. Although NIMH-funded policy research has experienced some growth during this time period, policy research remains a small share of NIMH's research portfolio. As more research highlights the importance of policies in shaping psychological health and well-being, it appears that NIMH funding may not be commensurate with this growing scientific understanding. If the mental health field persists in prioritizing the study of individual-level, largely biological, factors associated with mental illness at the expense of investigating structural factors, like policies, our collective understanding of and solutions to population mental health challenges will be incomplete.

## Supplementary Material

qxag099_Supplementary_Data
