# Supplementary material for: U.S. policy research funded by the National Institute of Mental Health, 1993-2024
Source: Health Aff Sch. 2026 Apr 29;4(5):qxag099. doi: 10.1093/haschl/qxag099 (PMC13186268; doi:10.1093/haschl/qxag099)
Supplement: qxag099_Supplementary_Data [file qxag099_supplementary_data.zip › nimh_appendix.docx]

**Appendix**

**Supplemental Figure 1.** Study Flow Diagram

**Excluded**:

Projects that did not fit operationalized definition of U.S. policy research from Title, Abstract, and Publications.

(total n = 6,044; new n = 1,548)

Projects with “policy” keywords (policy, policies, law, legal, waiver, payment, pay, reimbursement, Medicare, Medicaid, statute, “executive order,” and bill) in Title, Abstract, and Key Terms in NIH RePORTER

(total n = 7,538; new n = 1,823)

Projects that fulfilled the operationalized definition of U.S. policy research

(total n = 1,510; new n = 275)

**Supplemental Table 1.** National Institute of Mental Health (NIMH) funding for policy research by National Institutes of Health activity code, 1993-2024.

| **Activity Code** | **Total Projects (n)** | **Share of Total Policy Projects (%)** | **Total Projects Funding ($, unadjusted)** | **New Projects (n)** | **Share of New Policy Projects (%)** | **New Projects Funding ($, unadjusted)** |
| --- | --- | --- | --- | --- | --- | --- |
| R01 | 479 | 31.72% | $213,009,666 | 124 | 45.09% | $60,260,789 |
| T32 | 296 | 19.60% | $76,914,907 | 10 | 3.64% | $1,465,361 |
| P30 | 256 | 16.95% | $272,155,981 | 11 | 4.00% | $13,304,461 |
| P50 | 109 | 7.22% | $134,831,447 | 7 | 2.55% | $9,117,526 |
| K01 | 73 | 4.83% | $11,165,108 | 15 | 5.45% | $2,459,791 |
| U01 | 41 | 2.72% | $22,986,870 | 8 | 2.91% | $5,453,450 |
| R03 | 37 | 2.45% | $2,633,304 | 21 | 7.64% | $1,484,510 |
| R21 | 33 | 2.19% | $6,102,204 | 15 | 5.45% | $3,348,833 |
| R34 | 25 | 1.66% | $5,151,531 | 8 | 2.91% | $1,943,684 |
| R29 | 21 | 1.39% | $2,221,915 | 4 | 1.45% | $501,989 |
| R24 | 21 | 1.39% | $7,124,158 | 9 | 3.27% | $2,556,725 |
| F31 | 19 | 1.26% | $728,808 | 12 | 4.36% | $453,667 |
| K02 | 15 | 0.99% | $1,894,310 | 3 | 1.09% | $375,409 |
| K23 | 13 | 0.86% | $2,191,627 | 3 | 1.09% | $537,214 |
| P01 | 12 | 0.79% | $12,306,934 | 3 | 1.09% | $4,378,742 |
| K05 | 11 | 0.73% | $1,182,261 | 2 | 0.73% | $217,485 |
| F30 | 9 | 0.60% | $396,453 | 3 | 1.09% | $127,527 |
| U24 | 8 | 0.53% | $10,085,115 | 1 | 0.36% | $1,980,274 |
| RC1 | 6 | 0.40% | $2,474,794 | 3 | 1.09% | $1,258,578 |
| RF1 | 5 | 0.33% | $12,267,861 | 5 | 1.82% | $12,267,861 |
| R36 | 5 | 0.33% | $179,665 | 3 | 1.09% | $116,843 |
| K08 | 4 | 0.26% | $756,000 | 1 | 0.36% | $189,000 |
| F32 | 3 | 0.20% | $171,704 | 1 | 0.36% | $53,432 |
| UF1 | 2 | 0.13% | $0 | 1 | 0.36% | $0 |
| Not Classified | 2 | 0.13% | $0 | 0 | 0.00% | $0 |
| T34 | 1 | 0.07% | $274,145 | 0 | 0.00% | $0 |
| R61 | 1 | 0.07% | $0 | 1 | 0.36% | $0 |
| R44 | 1 | 0.07% | $998,118 | 0 | 0.00% | $0 |
| P20 | 1 | 0.07% | $387,764 | 0 | 0.00% | $0 |
| K18 | 1 | 0.07% | $0 | 1 | 0.36% | $0 |
| Grand Total | 1510 | 100.00% | $800,592,650 | 275 | 100.00% | $123,853,151 |

Note: Authors’ analysis of publicly available data from NIH RePORTER. Activity codes are identifiers that represent unique National Institutes of Health (NIH) funding programs. The NIH provides funding for a variety of research and research-related activities (including conferences, training institutes, predoctoral fellowships, etc.), each of which fall into different funding categories. The first letter of the activity code indicates the funding category. For example, activity codes that begin with “R” typically represent research and development grants and activity codes that begin with “K” typically represent career development grants. See the NIH website for the list of activity codes, their funding categories, and longer descriptions of their functions: <https://grants.nih.gov/funding/activity-codes>.

**Supplemental Figure 2.** New and total policy projects funded by the National Institute of Mental Health (NIMH) over time, 1993-2024


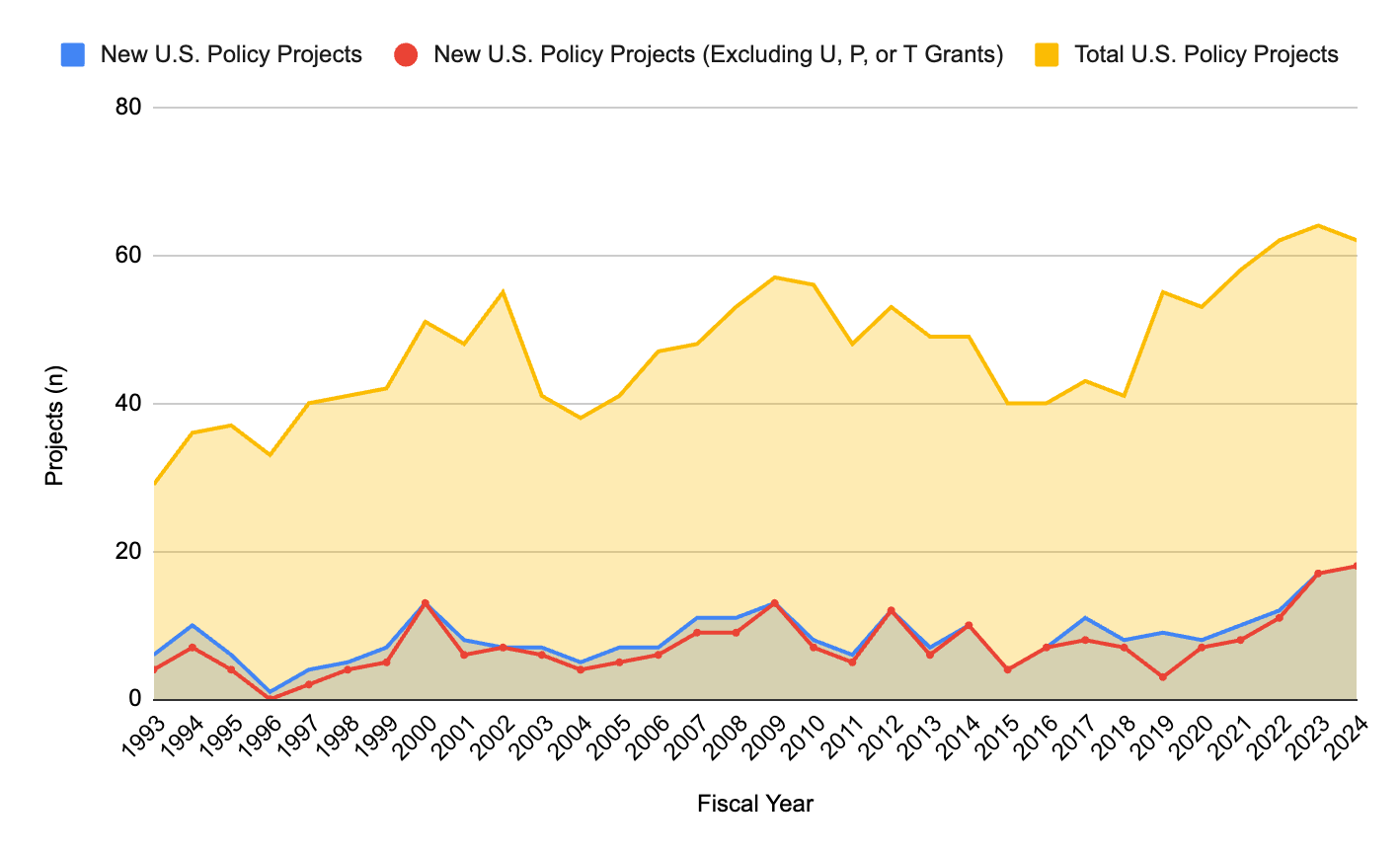


Note: U, P, T projects represent specific National Institutes of Health (NIH) grant types: Cooperative Agreements (U), Program Project/Center Grants (P), and Training Grants (T)

**Supplemental Figure 3.** National Institute of Mental Health (NIMH) funding for policy research over time in 2024-adjusted dollars, 1993-2024.


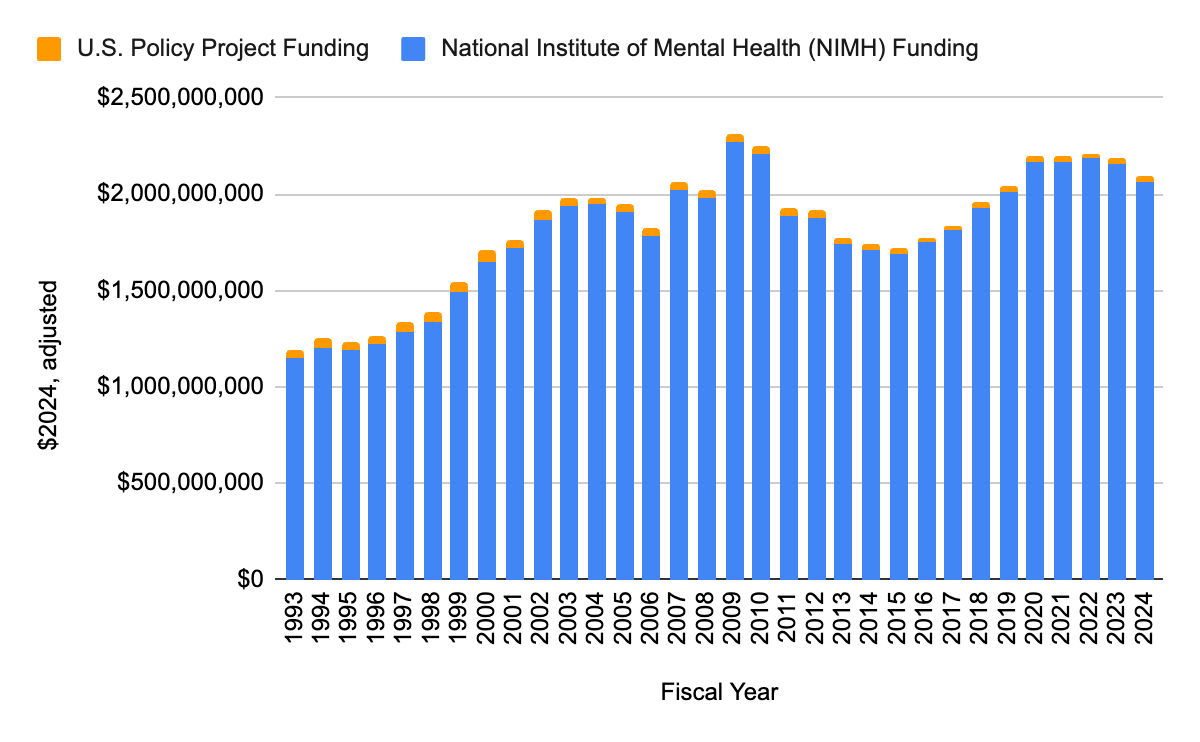


**Supplemental Figure 4.** New and total policy projects funded by the National Institute of Mental Health (NIMH) over time in 2024-adjusted dollars, 1993-2024


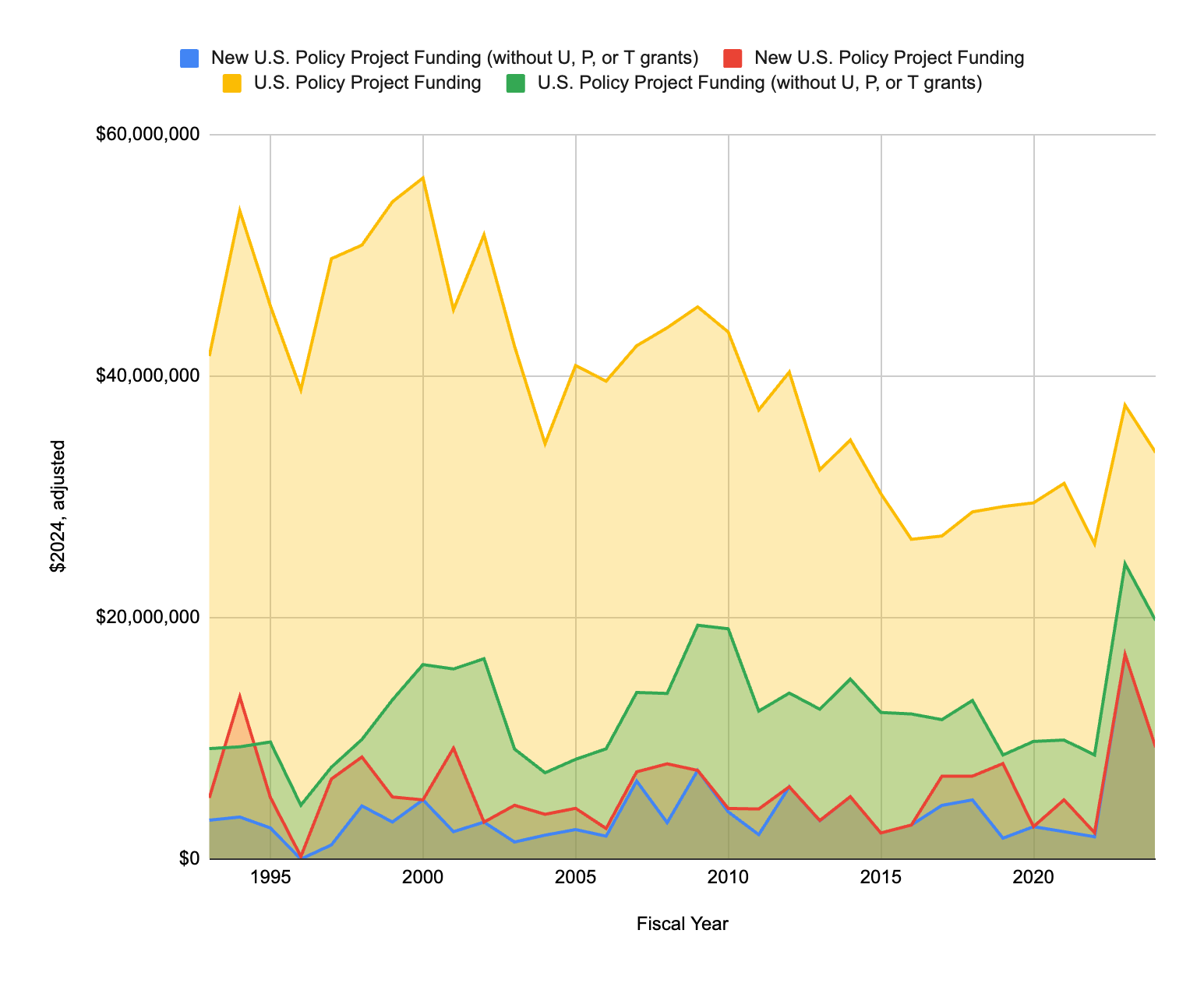


Note: U, P, T projects represent specific National Institutes of Health (NIH) grant types: Cooperative Agreements (U), Program Project/Center Grants (P), and Training Grants (T)
