# Supplementary material for: U.S. policy research funded by the National Institute of Mental Health, 1993-2024
Source: Health Aff Sch. 2026 Apr 29;4(5):qxag099. doi: 10.1093/haschl/qxag099 (PMC13186268; doi:10.1093/haschl/qxag099)
Supplement: qxag099_Supplementary_Data [file qxag099_supplementary_data.zip › coi_disclosure.docx]

| ICMJE DISCLOSURE FORM | |
| --- | --- |
| **Date:** | 4/17/2026 |
| **Your Name:** | Briana S. Last, Nicole Teow, Madeline Poupard |
| **Manuscript Title:** | U.S. Policy Research Funded by the National Institute of Mental Health, 1993-2024 |
| **Manuscript Number (if known):** | Click or tap here to enter text. |
| In the interest of transparency, we ask you to disclose all relationships/activities/interests listed below that are related to the content of your manuscript. “Related” means any relation with for-profit or not-for-profit third parties whose interests may be affected by the content of the manuscript. Disclosure represents a commitment to transparency and does not necessarily indicate a bias. If you are in doubt about whether to list a relationship/activity/interest, it is preferable that you do so.  The author’s relationships/activities/interests should be defined broadly. For example, if your manuscript pertains to the epidemiology of hypertension, you should declare all relationships with manufacturers of antihypertensive medication, even if that medication is not mentioned in the manuscript.  In item #1 below, report all support for the work reported in this manuscript without time limit. For all other items, the time frame for disclosure is the past 36 months. | |

|  | | | **Name all entities with whom you have this relationship or indicate none (add rows as needed)** | **Specifications/Comments (e.g., if payments were made to you or to your institution)** |
| --- | --- | --- | --- | --- |
| **Time frame: Since the initial planning of the work** | | | | |
| **1** | All support for the present manuscript (e.g., funding, provision of study materials, medical writing, article processing charges, etc.)  **No time limit for this item.** | | \|  \| **None** \| \| --- \| --- \|  \| Dr. Last is currently a National Institute of Mental Health (NIMH) Implementation Research Institute Fellow (R25MH080916). \|  \| \| --- \| --- \| \|  \|  \| \|  \| Click the tab key to add additional rows. \| | |
| **Time frame: past 36 months** | | | | |
| **2** | | Grants or contracts from any entity (if not indicated in item #1 above). | \|  \| **None** \| \| --- \| --- \|  \| Dr. Last has received grant funding from the American Psychological Foundation, the Association for Clinical Psychological Science, Stony Brook University, the AIM Youth Mental Health Foundation, and NIMH. \|  \| \| --- \| --- \| \|  \|  \| \|  \|  \| | |
| **3** | | Royalties or licenses | \|  \| **None** \| \| --- \| --- \|  \|  \|  \| \| --- \| --- \| \|  \|  \| \|  \|  \| | |
| **4** | | Consulting fees | \|  \| **None** \| \| --- \| --- \|  \| Dr. Last received consulting fees from MDRC in 2024 and the Yale LGBTQ+ Mental Health Initiative in 2026 \| Payments made to me \| \| --- \| --- \| \|  \|  \| \|  \|  \| \|  \|  \| | |
| **5** | | Payment or honoraria for lectures, presentations, speakers bureaus, manuscript writing or educational events | \|  \| **None** \| \| --- \| --- \|  \| Dr. Last has received honoraria from the International Society for the Improvement and Teaching of Dialectical Behavior Therapy, the Association for Behavioral Cognitive Therapies, and Northwestern Feinberg School of Medicine for presenting research. \|  \| \| --- \| --- \| \|  \|  \| \|  \|  \| | |
| **6** | | Payment for expert testimony | \|  \| **None** \| \| --- \| --- \|  \|  \|  \| \| --- \| --- \| \|  \|  \| \|  \|  \| | |
| **7** | | Support for attending meetings and/or travel | \|  \| **None** \| \| --- \| --- \|  \| Dr. Last received support for attending the International Society for the Improvement and Teaching of Dialectical Behavior Therapy annual meeting. \|  \| \| --- \| --- \| \|  \|  \| \|  \|  \| | |
| **8** | | Patents planned, issued or pending | \|  \| **None** \| \| --- \| --- \|  \|  \|  \| \| --- \| --- \| \|  \|  \| \|  \|  \| | |
| **9** | | Participation on a Data Safety Monitoring Board or Advisory Board | \|  \| **None** \| \| --- \| --- \|  \|  \|  \| \| --- \| --- \| \|  \|  \| \|  \|  \| | |
| **10** | | Leadership or fiduciary role in other board, society, committee or advocacy group, paid or unpaid | \|  \| **None** \| \| --- \| --- \|  \|  \|  \| \| --- \| --- \| \|  \|  \| \|  \|  \| | |
| **11** | | Stock or stock options | \|  \| **None** \| \| --- \| --- \|  \|  \|  \| \| --- \| --- \| \|  \|  \| \|  \|  \| | |
| **12** | | Receipt of equipment, materials, drugs, medical writing, gifts or other services | \|  \| **None** \| \| --- \| --- \|  \|  \|  \| \| --- \| --- \| \|  \|  \| \|  \|  \| | |
| **13** | | Other financial or non-financial interests | \|  \| **None** \| \| --- \| --- \|  \| Ms. Teow is currently a product manager at Teladoc; she worked on this manuscript as a research assistant at Stony Brook University prior to initiating her role. Prior to working on this manuscript, Ms. Teow was employed at Tia, Inc in product and partnership roles. Ms. Poupard is currently a part-time employee at the Center for Motivation and Change. Ms. Poupard completed this manuscript as a part-time research assistant at Stony Brook University. Both Ms. Teow and Ms. Poupard’s current and previous employment relationships have no bearing on the submitted work. The manuscript does not represent the views, perspectives, or opinions of any of the authors’ current or former employers. \|  \| \| --- \| --- \| \|  \|  \| \|  \|  \| | |
|  | |  |  | |
| **Please place an “X” next to the following statement to indicate your agreement:** | | | | |
|  | | I certify that I have answered every question and have not altered the wording of any of the questions on this form. | | |
